# The Cause of Necrosis under Silicate Cements

**Published:** 1913-05-15

**Authors:** 


### THE CAUSE OF NECROSIS UNDER SILICATE CEMENTS.

Dr. Proell, speaking on the results obtained in the bacteriological division of the 1st Army Corps Institute of Hygiene, drew attention to the fact that while the death of the pulp under all kinds of fillings is popularly attributed either to careless or insufficient preparation of the cavity or (in cases where every care has been taken) sometimes, to the action of poisons contained in the filling materials—there are so far to hand no reliable results of research on this particular question of the death of the pulp under silicate cements.

At the instigation of Professor Romer of Strassburg, who is of opinion that the pulp dies not in consequence of chemico-toxic processes, but because injurious substances find their way through the pores of the filling or by some other means and reach the hitherto untouched pulp, Dr. Proell undertook a long series of experiments and observations, amongst which were included the use of various fillings at many temperatures and degrees of fluidity and the close observation of their behavior and their various modifications in size, weight, porosity to certain fluids, etc. His general conclusions are as follows:—

1. The death of the pulp under zinc-phosphate and silicate cement fillings is due less to a chemico-toxic action than to an infection and is similar in its clinical phenomena to the gangrene, with a subsequent ostitis and a purulent periostitis, of a hidden caries.

2. The moment of infection is not due to the failure of an antiseptic process—freshly-made cements have, on the contrary, the least power of arresting growths.

3. Nor is it that the materials used favor the entrance of bacteria.

4. A certain amount of moisture does, no doubt, penetrate the cement, but neither water nor ether can be proved, experimentally, to get through.

5. Many silicate and zinc-phosphate cements exhibit in the mouth certain modifications; primarily a shrinkage disturbing, to some extent, the edges of the filling. This change of bulk is, most probably, the cause of the gangrene of a pulp under a cement filling.

6. Exposed to the air both a new and an old silicate cement filling quickly dry and contract.

7. (*a*) This peculiarity makes such materials unsuitable for filling teeth—especially the front teeth—which are, for whatever reason, not continually moistened by the saliva. (*b*) In each case the cavity must be most scrupulously prepared and a perfect adhesion must be ensured by means of some substance which shall adhere to the walls of the cavity in spite of the contracting edges of the filling. (*c*) Any drying of fresh or old silicate cement fillings must be most carefully avoided.

8. The efficacy of the two prevalent methods of securing adhesion, *i. e.*, the varnishing of the walls or the making of a foundation with zinc-phosphate cement, is not borne out by experiment.

9. The various silicate cements used in dentistry are, in their consistency, not unlike Portland cement, but it may be gathered from the foregoing experiments that they are, as a matter of fact, chemically speaking, nearer to zinc-phosphate cements that to hydraulic mortar.—*Deutsche Monatsschrift fuer Zahnheilkunde.*

